# Designing and Expression of Recombinant Chimeric Spike Protein from SARS-CoV-2 in *Escherichia coli* and Its Immunogenicity Assessment

**DOI:** 10.5812/ijpr-137751

**Published:** 2023-09-10

**Authors:** Sahar Karimi, Shahram Nazarian, Fattah Sotoodehnejadnematalahi, Roohollah Dorostkar, Jafar Amani

**Affiliations:** 1 Department of Biology, Science and Research Branch, Islamic Azad University, Tehran, Iran; 2 Department of Biology, Imam Hossein University, Tehran, Iran; 3 Applied Virology Research Center, Baqiyatallah University of Medical Sciences, Tehran, Iran; 4 Applied Microbiology Research Center, System Biology and Poisonings Institute, Baqiyatallah University of Medical Sciences, Tehran, Iran

**Keywords:** Bioinformatics, Neutralizing Antibody, Vaccines, SARS-CoV-2, Spike Protein

## Abstract

Since December 2019, the world has been grappling with an ongoing global COVID-19 pandemic. Various virus variants have emerged over the past two years, each posing a greater threat than its predecessors. The recent appearance of the omicron variant (B.1.1.529) has raised significant alarm within the field of epidemiology due to its highly contagious nature and rapid transmission rate. The omicron variant possessed mutations in the key receptor-binding domain (RBD) region, the S region, and these modifications have shown a notable impact on the strain's susceptibility to neutralizing antibodies. Developing safe and efficient vaccines to prevent a future severe acute respiratory outbreak of coronavirus syndrome 2 (SARS-CoV-2) is significant. Viral surface spike proteins are ideal targets for vaccines. This study aimed to find a multi-subunit chimeric vaccine. After conducting bioinformatics analysis, the recombinant spike (RS) protein of SARS-CoV-2 was deliberately designed and subsequently produced using *E. coli* expression systems. The immunogenicity of RS and neutralizing antibody responses were evaluated on immunized BALB/c mice. There was a significant difference in antibody titers between RS-immunized mice and control groups. The endpoint of the serum antibody titer of mice immunized with our chimeric protein was 2.5 times higher than that of the negative control. The chimeric construct could present multiple antigens simultaneously, influentially affecting immunization. Sera from mice vaccinated by RS could recognize the SARS-CoV-2 virus and neutralize antibodies. Our chimeric peptide could bind to antibodies in the serum of patients infected with different serotypes of the SARS-CoV-2 virus, such as alpha, delta, and omicron variants. The results indicated that the RS protein would be a potential novel antigenic candidate for subunit vaccine development and could be used as a useful alternative to generate diagnostic serological tests for SARS-CoV-2 infection.

## 1. Background

Originating in Wuhan, China, the SARS-CoV-2 virus, a member of the beta-coronavirus family, swiftly emerged as the causative agent behind the global COVID-19 disease, spreading rapidly across continents and impacting numerous countries worldwide (1-3). Although COVID-19 is not fatal at a young age, mortality increases exponentially with age. Men have a higher mortality rate than women, especially those with age-related diseases such as diabetes and hypertension (4). This new virus is more similar to SARS-CoV than to the MERS coronavirus containing 380 amino acid substitutions in the encoded proteins (5).

Studies of SARS-CoV-1 and dependent MERS-CoV vaccines have revealed that the spike protein on the virus's surface is an appropriate target for the vaccine (6). The S-protein can induce neutralizing antibodies (nAbs) and protective immunity among all other structural proteins (7). Spike contributes to cell tropism and virus entry (8). It can be recognized directly by the host immune system (1). The S-protein has two functional sub-units for binding to the host cell receptor (S1 subunit) and fusion of the cellular and viral membranes (S2 subunit) (9). The S1-protein has a conserved receptor-binding domain (RBD) that detects angiotensin-converting enzyme 2 (ACE2) in the human cell as a receptor (10), while the S2-protein contains fusion peptide (FP) (11). The viral spike protein carries the S1/S2 cleavage site. The cleavage of the spike protein at the S1/S2 site by the cellular furin protease plays a crucial role in facilitating the invasion of human lung cells and cell-cell fusion mediated by the S-protein (12).

The new variants of SARS-CoV-2 were detected via sequencing and based on the spike (S) gene status (13). Since the onset of the pandemic, SARS-CoV-2 has been undergoing continuous mutations. As an RNA virus, it possesses a high mutation rate, short replication time, and relatively low genome stability. These characteristics enable the virus to mutate rapidly, spread quickly, adapt to new environmental conditions, and maintain its genetic material, leading to evolutionary changes. Consequently, new SARS-CoV-2 strains with distinct genomic characteristics, varying levels of infection severity, and immune evasion have emerged. Notable mutant strains include alpha (B.1.1.7), beta (B.1.351), gamma (P.1), delta (B.1.617.2), lambda (C.37), mu (B.1621), eta (B.1.525), iota (B.1.526), kappa (B.1.617.1), and the more recent omicron (B.1.1.529). These variants are categorized into three groups: Variants of concern (VOC), variants of interest (VOI), and variants under monitoring (VUM).

Omicron, first reported to the World Health Organization (WHO) in South Africa on November 24, 2021, has exhibited significant modifications in its S protein with 26 - 32 protein S alterations. These modifications have notably impacted the strain's susceptibility to neutralizing antibodies. In comparison, the delta variant had only five mutations in the RBD, contributing to increased viral entry into human cells and vaccine-induced immunity evasion (14). A widespread worldwide serological test is required for SARS-CoV-2 to indicate the virus exposure extent in a certain area, the extent and duration of immunity after being infected, and the ratio of symptomatic to asymptomatic infected people.

In this regard, developing cost-effective and reliable tests for the timely diagnosis of antigens and producing safe and efficient vaccines for preventing a future SARS-CoV-2 epidemic is critical (15). Inactivated or attenuated pathogens are utilized traditionally as vaccines, while recombinant protein vaccines, especially chimeric ones, have benefits for expressing, cloning, and purifying genes from different etiological agents to be evaluated as vaccines. The chimeric construct can present multiple antigens simultaneously, influentially affecting immunization.

## 2. Objectives

In the present study, we selected SARS-CoV-2 viral epitopes with antigenic properties that play a major role in viral entry and pathogenesis. After bioinformatics analysis, a chimeric construct with RBD, S1/S2 cleavage, and FP was designed and expressed in *E. coli* (recombinant spike (RS)-Ecoli) to investigate its capacity for immunogenicity as a vaccine. Finally, RS can be considered a potential subunit vaccine against SARS-CoV-2 and an antigen for diagnostic serological tests for patients infected with COVID-19.

## 3. Methods

### 3.1. Bioinformatics Construct Design

To design the recombinant construct, the gene sequences of the surface glycoprotein (S (National Center for Biotechnology Information (NCBI) reference sequence: YP_009724390.1)) of SARS-CoV-2 were obtained from the NCBI (16). Online signal peptide prediction software was employed to identify and mark the signal peptide regions within the sequences (Table 1) (17). The DNA encoding a histidine (6-mer) sequence was also appended to enable carboxy-terminal expression in the construct (18). The chimeric construct comprises the RBD fused with the S1/S2 cleavage site connected to the FP protein. The constructed protein's secondary structure was determined by the Prabi server at SOPMA (19). The 3D structure of the chimeric protein was generated using the Swiss Model server and further optimized using Discovery Studio software (Accelrys) for enhanced structural refinement (20). Coordinates were prepared by uploading 3D structures in PDB format into PROSA-web to detect the errors in the produced models, often used to validate protein structures (21). The structure was validated in PROCHECK software by Ramachandran plot to observe the resulting stereochemistry quality (22). The RNA mfold program was used to examine the structural properties like RNA stability (23). The RS structure was investigated to identify Tcell epitopes. The MHC-I restricted epitopes were predicted using IEDB (24). The conformational B-cell epitopes in the vaccine model were predicted using the ElliPro server with epitope prediction parameters (25), employing a minimum score of 0.5 and a maximum distance of 6 angstroms. Furthermore, amino acid composition and some physicochemical properties of the constructed protein, including molecular weight, isoelectric point, net charge at pH 7, half-life in the mammalian reticulocytes, and instability index, were estimated using Protparam (26). Chimer protein was further evaluated regarding antigenicity, allergenicity, toxicity, and solubility using bioinformatics tools in Table 1. To facilitate the cloning and expression of the proposed candidate vaccine in a suitable expression vector, the amino acid sequence of the vaccine model was back-translated into nucleotide sequences using the Gene Infinity server based on the codon usage table of *E. coli* (27). The resulting DNA sequence was then optimized by assessing its GC content, codon adaptation index (CAI), and codon frequency distribution (CFD) using the GenScript Tool (20). These parameters are crucial in optimizing the host system's protein expression. Subsequently, the DNA sequence was examined for restriction sites using NEB Cutter V2.0 to map the locations of potential restriction sites (28). Proper restriction sites were then added to the 5' and 3'-OH ends of the optimized DNA sequence to facilitate subsequent cloning steps (20).

**Table 1.** Used Bioinformatics Tools for Silico Analyses

Tool	Short Description	References
**SignalP 4.0**	A software tool for predicting signal peptide sequences and their cleavage positions in bacterial and eukaryotic proteins	(17)
**Prabi**	A predicting tool for protein secondary structure	(19)
**Swiss Model**	A server for predicting protein structure homology-modeling	(20)
**PROSA-web**	An interactive web service for the recognition of errors in three-dimensional structures of proteins	(21)
**PROCHECK**	A program to check the stereochemical quality of protein structures	(22)
**IEDB**	An immunoinformatics tool for the prediction of the population coverage of an epitope	(25)
**ProtParam**	Investigation of physicochemical characteristics of the protein	(26)
**VaxiJen**	A web-based tool for the prediction of protein antigenicity	(20)
**AlgPred**	A predicting tool for the determination of allergenicity of a protein	(20)
**Toxin pred**	Prediction of the toxic regions in a protein sequence	(20)
**PepCalc**	An online tool for the prediction of protein solubility from its sequence	(20)
**Infinity **	An in-silico tool for genome-wide prediction of specific DNA matrices in miRNA genomic loci	(27)
**NEB cutter**	A program to cleave DNA with restriction enzymes	(28)

### 3.2. Cloning the Constructs

To amplify the chimeric gene, specific primers 5′ ATATATGAAT TCGCCACCATGGTGAGGGT 3′ (forward) and 5′ ATAGATAAGCTTCAGGCCGTTGAACTTCTGG 3′ (reverse) were developed.

For PCR amplification of the targeting, the RS region was utilized. The resulting PCR product was subjected to digestion using the Rapid Digest *Eco*RI and *Hin*dIII enzymes. Subsequently, the digested PCR product was ligated into the pET-28a+ vector that was similarly digested with *Eco*RI and *Hin*dIII, employing T4 ligase from Fermentas, Germany. This ligation step generated the pET-28a-rs (pET-*rs*) construct. The pET-*rs* construct was then transformed into competent *E. coli* Rosetta-gami (DE3) cells. These cells were cultured on Luria-Bertani (LB) agar plates supplemented with 50 μg/mL kanamycin to enable the selection of transformed cells. The resulting colonies were further analyzed through restriction digestion and colony PCR techniques to confirm the successful insertion of the desired construct.

### 3.3. Preparation of Recombinant RS Protein

A colony containing the pET-*rs* construct was grown in LB medium until it reached an optical density of 0.7. Optimal induction of the recombinant protein was achieved by adding 1 mM isopropylthio-β-D-galactoside (IPTG, Fermentas), followed by incubation for 16 hours at 25°C. Non-induced cells harboring the recombinant plasmid were used as a negative control in the experiment.

The chimera protein was subsequently purified under denaturing conditions using immobilized metal affinity chromatography (IMAC) with Ni-NTA agarose (Qiagen). The purification process involved running the protein samples through an SDS-PAGE gel with a concentration of 12%. Western blotting analysis was performed to confirm the expression of the RS protein.

### 3.4. Western Blotting

To separate the RS protein, SDS-PAGE with a concentration of 12% was employed. The protein bands were then electro-transferred from the gel onto a PVDF membrane (Roche). Following the transfer, the PVDF membrane was subjected to blocking using 5% non-fat skim milk in TBS buffer (50 mM Tris-Cl, 150 mM NaCl, pH 7.5) containing 0.05% Tween 20. The blocking step was carried out at 37°C for 2 hours. For protein detection, the PVDF membrane was incubated with HRP-conjugated mouse anti-poly His-tag antibody (1:2000, Roche). Subsequently, the membrane was immersed in a solution containing 3,3'-Diaminobenzidine (DAB Reagents, Sigma) to develop the signal and visualize the protein bands on the membrane.

### 3.5. Mice Immunization

All experimental procedures in this study adhered to international ethical standards and were approved by the Ethics Committee of Islamic Azad University-Science and Research Branch (approval number: 2021-06-16/IR.IAU.SRB.REC.1400.075). A total of 20 BALB/c mice (female, 6 - 7 weeks old, weighing 20 - 25 g) were obtained from the Razi Institute in Tehran, Iran. The mice were divided into two groups (10 mice per each group), namely the test group and the control group. All mice in the test group were first vaccinated subcutaneously with 15 μg protein (75 μL RS protein with 75 μL PBS) mixed with an equal volume of complete Freund’s adjuvant. The second and third doses were provided subcutaneously as boosters of 15 μg protein with incomplete Freund's adjuvant (at 15-day intervals). Also, PBS with adjuvant was used for the control group. Blood samples were collected from the eye corners of the mice on days 29 and 44 using capillary tubes (3, 23, 29). The collected blood samples were centrifuged to obtain serum, which was then stored at -20°C until further use. All individual serum samples were pooled together for subsequent immunological analyses.

### 3.6. Investigation of Serum IgG Titer

The IgG levels in the mouse sera were determined using an indirect enzyme-linked immunosorbent assay (ELISA). In each well of an ELISA plate, 1 μg of RS protein (dissolved in a coating buffer consisting of 35 mM NaHCO3, 15 mM Na_2_CO_3_, pH 9.5) was added in a volume of 100 μL and incubated at 4°C overnight. The plate wells were then washed with 0.05% phosphate-buffered saline with 0.05% Tween 20 (PBST). A blocking buffer containing PBST and 5% skimmed milk powder were added to the wells and incubated at 37°C for 1 hour to block non-specific binding sites. The plate wells were washed with PBST to remove any unbound substances. Serial dilutions of the antibody serum, ranging from 1:100 to 1: 256,000 in PBST, were added to each well. The plate was incubated at 37°C for 2 hours, followed by another wash round with PBST. Next, a goat anti-mouse IgG HRP conjugate from Sigma Company (diluted 1:2000 in PBST) was added to each well and incubated at 37°C for 1.5 hours. After washing the plate with PBST, 100 μL of 3,3',5,5'-tetramethylbenzidine (TMB) substrate solution (0.4 g/L) was added to each well. The reaction was stopped by adding 2M H_2_SO_4_, and the absorbance of the plate was read at 450 nm.

### 3.7. Recognition of Inactivated SARS-CoV-2 by Serum of Mice Immunized with RS

To do so, 10^6^ pfu of the SARS-CoV-2 virus, which was cultured in vero cells and inactivated using 4% paraformaldehyde, accumulated at a concentration of 100 μg from the Pasteur Institute of Iran. After in vitro optimization, a concentration of 1000 ng of the inactivated virus was chosen for the ELISA test. The Dulbecco's modified eagle medium (DEME) medium, containing Vero cells, was considered a negative control. Also, 1000 ng of inactivated SARS-CoV-2 was coated in 100 μL of ELISA coating buffer on an ELISA plate. Serial dilution of antibody serum of mice immunized from 1:1 to 1:100000 in PBST was added to every well.

### 3.8. ELISA Test for Examining the Immunogenicity of RS as a Vaccine

To evaluate the immunogenicity of the RS protein as a vaccine for humans, serum samples were collected from 50 COVID-19 convalescent participants infected with the SARS-CoV-2 alpha variant, 50 COVID-19 convalescent participants infected with the SARS-CoV-2 delta variant, 50 COVID-19 convalescent participants infected with the SARS-CoV-2 omicron variant, and 20 healthy donors. The ELISA was performed to assess the binding of serum antibodies to RS, following the previously described method (section 2.6.). In brief, 96-well microtiter plates were coated with RS protein, and a serial dilution of antibody serum ranging from 1:1 to 1: 100,000 in PBST was added to each well after blocking with 5% skim milk. The plates were then incubated at room temperature for 1 hour, followed by four washes with PBST. The bound antibodies were detected using goat anti-human IgG antibodies conjugated with horseradish peroxidase (Sigma).

### 3.9. Investigation of Sera from Immunized Mice for the Blocking Activity of the RBD Binding to ACE2 Receptor

According to the manufacturer's guidelines, serum from immunized animals was tested to block the activity of RBD binding to the ACE2 receptor using the SARS-CoV-2 neutralizing antibody ELISA test kit (Pishtaz Teb, Iran).

### 3.10. Statistical Analyses

Statistical analysis was performed using SPSS 24 software to compare the antibody responses obtained from the immunized and unvaccinated groups and the levels of SARS-CoV-2 neutralizing antibodies. One-way ANOVA was employed for this analysis. A significance level of P < 0.05 was used to determine if the observed differences were statistically significant.

## 4. Results

### 4.1. Bioinformatics Construct Design

**Figure 1.**
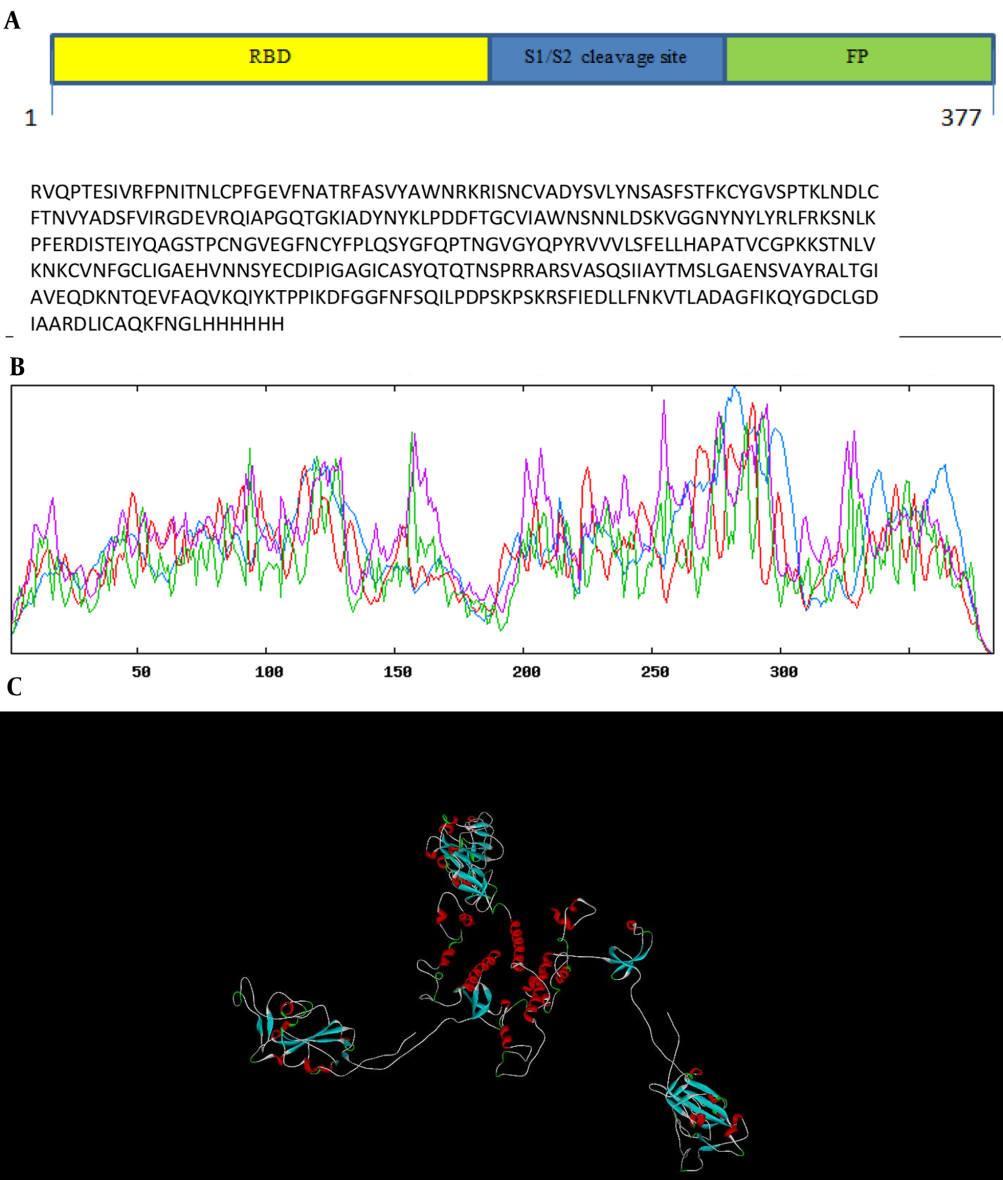
The recombinant spike (RS) construct structure. A, schematic diagram of RS construct containing RBD, domain S1/S2 cleavage, and fusion peptide (FP) protein; B, graphical results for secondary structure prediction. Alpha helix (blue): 22.72%, extended strand (red): 21.15%, random coil (yellow): 47.78%; C, 3D structure of RS predicted by Swiss model server.

**Figure 2.**
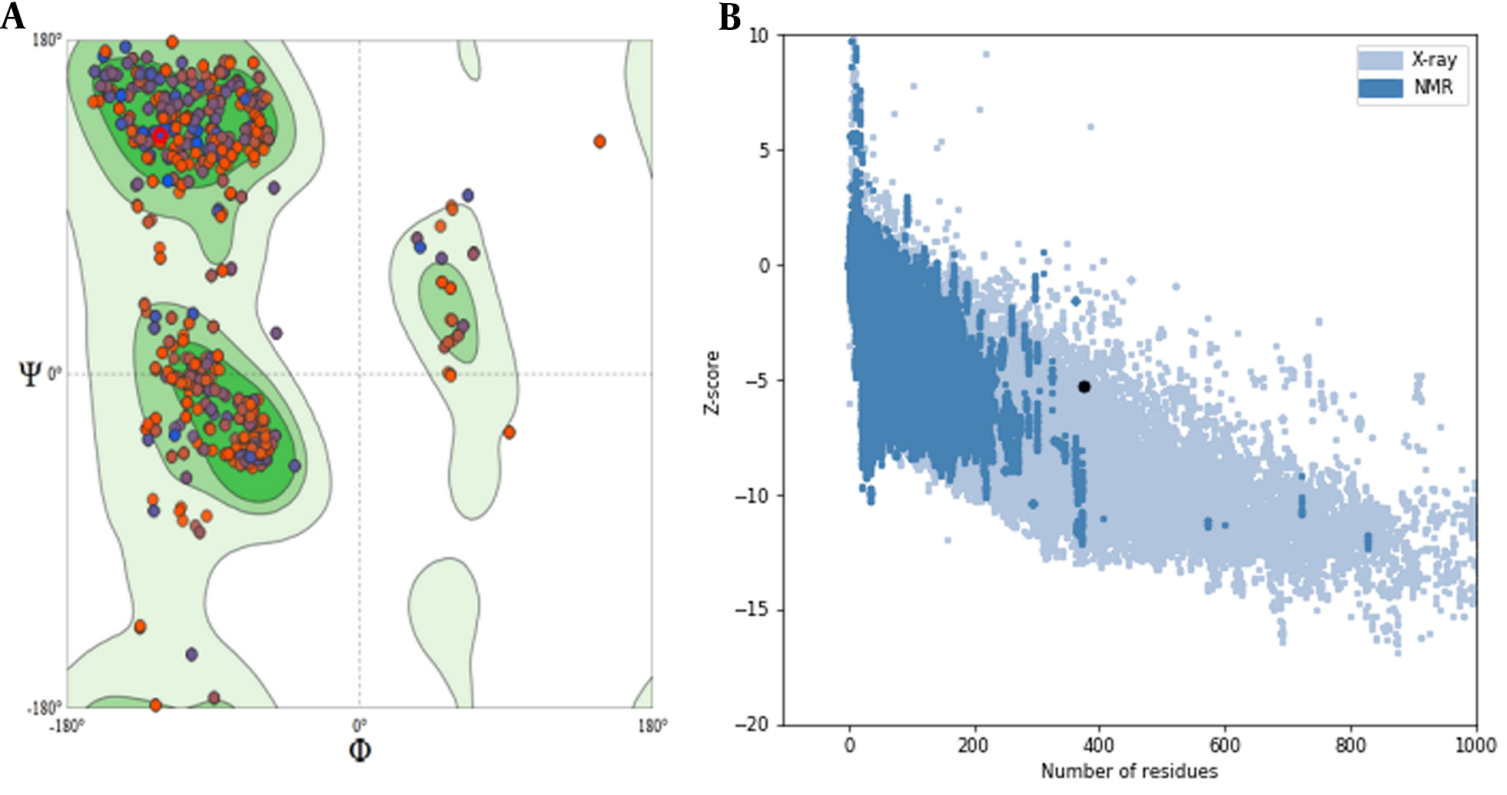
A, evaluation of model stability is based on a Ramachandran plot for the RS chimeric protein. The percentage of the residue was 92.69% in the favored region, 1.78% allowed, and 5.53% outlier; B, ProSA Z-score indicated that the 3D structure of the vaccine is of high quality.

The gene encoding sequences of SARS-CoV-2 surface glycoprotein (S (NCBI reference sequence:-YP_009724390.1)) were obtained from the NCBI database, and the complete RS sequence was designed (Figure 1A). The physicochemical properties of the vaccine construct were evaluated (Table 2). To create a potential vaccine, the sequence of amino acids was converted into a sequence of nucleotides and then optimized for efficient protein expression. The gene's key properties contributing to high protein expression were also predicted. The optimized gene has a CAI value of 0.7, which is ideal for expression in the desired organism, and an average GC content of 49.64%, falling within the optimal range of 30 - 70%. The gene's CFD in *E. coli* was 8%, indicating that it is suitable for cloning and expression. To clone the gene in *E. coli*, recognition sites for the EcoRI and HindIII restriction enzymes were added to the 5' and 3'-OH ends of the optimized gene, respectively. The secondary structure prediction (Figure 1B) revealed that the percentages of alpha-helices, extended strands, and random coils were 22.72%, 21.15%, and 47.78%, respectively. The 3D structure of the protein was generated using the Swiss model server (Figure 1C), and its quality was assessed through a Ramachandran plot and ProSA Z-score analysis. The Ramachandran plot demonstrated that the percentage of the residue was 92.69% in the favored region, 1.78% allowed, and 5.53% outlier region (Figure 2A). Additionally, the model's Z-score was -5.3, which falls within the range of scores typically observed for native proteins of similar sizes (21) (Figure 2B). These results indicate that the 3D model of the vaccine exhibits acceptable geometry and can be considered a reliable model for further evaluations. Predicting linear epitopes can be accomplished by considering various features such as hydrophobicity, flexibility, and surface properties and employing bioinformatics algorithms that leverage these characteristics (Table 3). The results of conformational B-cell epitope prediction are presented in Table 4. The results of T-cell epitope prediction are shown in Table 5. The RS molecular weight and PI were 42.6212 kDa and 8.65, respectively. There were 31 negatively charged amino acids (glutamic acid, aspartic acid) and 38 positively charged amino acids (lysine arginine). The extinction coefficient at 280 nm was 43165. The chimer aliphatic index, instability index, and grand average of hydropathicity (GRAVY) were 75.61, 29.92, and -0.261, respectively (Table 2). The instability index provides insight into the stability of the recombinant protein. Proteins with an instability index below 40 are considered stable (23). The primer sequences were designed after analyzing the sequence using OligoAnalyzer software (30). The *Hin*dIII and *Eco*RI restriction enzymes were utilized in the analysis.

**Table 2.** Physiochemical Properties of the Vaccine Construct

Protein	GC Content	CAI	CFD	Mw	TpI	-R	+R	EC	II	AI	GRAVY
**RS**	49.64%	0.7	8%	42.62	8.65	31	38	43165	29.92	75.61	-0.261

Abbreviations: RS, recombinant spike; GC content, guanine, and cytosine content; CAI, codon adaptation index; CFD, codon frequency distribution; Mw, molecular weight; TpI, theoretical isoelectric point; -R, number of negatively charged residues; +R, number of positively charged residues; EC, extinction coefficient at 280 nm; II, instability index; AI, aliphatic index; GRAVY, grand average hydropathy.

**Table 3.** Results of Linear B-cell Epitope Prediction ^a^

No.	Chain	Start	End	Peptide	Number of Residues	Score
**1**	A	77	197	VYADSFVIRGDEVRQIAPGQTGKIADYNYKLPDDFTGCVIAWNSNNLDSKVGGNYNYLYRLFRKSNLKPFERDISTEIYQAGSTPCNGVEGFNCYFPLQSYGFQPTNGVGYQPYRVVVLSF	121	0.833
**2**	A	12	70	PNITNLCPFGEVFNATRFASVYAWNRKRISNCVADYSVLYNSASFSTFKCYGVSPTKLN	59	0.695
**3**	A	204	212	ATVCGPKKS	9	0.528
**4**	A	259	264	RARSVA	6	0.518
**5**	B	116	141	IAWNSNNLDSKVGGNYNYLYRLFRKS	26	0.786
**6**	B	146	195	FERDISTEIYQAGSTPCNGVEGFNCYFPLQSYGFQPTNGVGYQPYRVVVL	50	0.775
**7**	B	5	58	TESIVRFPNITNLCPFGEVFNATRFASVYAWNRKRISNCVADYSVLYNSASFST	54	0.687
**8**	B	252	266	TQTNSPRRARSVASQ	15	0.594
**9**	B	305	321	KQIYKTPPIKDFGGFNF	17	0.583
**10**	B	203	218	PATVCGPKKSTNLVKN	16	0.567
**11**	B	103	106	YNYK	4	0.535
**12**	B	326	334	PDPSKPSKR	9	0.513
**13**	C	114	196	CVIAWNSNNLDSKVGGNYNYLYRLFRKSNLKPFERDISTEIYQAGSTPCNGVEGFNCYFPLQSYGFQPTNGVGYQPYRVVVLS	83	0.855
**14**	C	12	60	PNITNLCPFGEVFNATRFASVYAWNRKRISNCVADYSVLYNSASFSTFK	49	0.737
**15**	C	75	108	TNVYADSFVIRGDEVRQIAPGQTGKIADYNYKLP	34	0.675
**16**	C	314	319	KDFGGF	6	0.515
**17**	C	204	212	ATVCGPKKS	9	0.506

^a^ ElliPro predicts linear antibody epitopes based on a protein sequence. ElliPro accepts an input protein structure in PDB format. ElliPro associates each predicted epitope with a score, defined as a protrusion index (PI) value averaged over epitope residues. In the method, the protein's 3D shape is approximated by a number of ellipsoids; thus, the ellipsoid with PI = 0.9 would include within 90% of the protein residues with 10% of the protein residues being outside of the ellipsoid, while the ellipsoid with PI = 0.8 would include 80% of residues with 20% being outside the ellipsoid.

**Table 4.** Results of Discontinuous Epitope Prediction ^a^

No.	Residues	Number of Residues	Score
**1**	A:V9, A:F11, A:P12, A:N13, A:I14, A:T15, A:N16, A:L17, A:C18, A:P19, A:F20, A:G21, A:E22, A:V23, A:F24, A:N25, A:A26, A:T27, A:R28, A:F29, A:A30, A:S31, A:V32, A:Y33, A:A34, A:W35, A:N36, A:R37, A:K38, A:R39, A:I40, A:S41, A:N42, A:C43, A:V44, A:A45, A:D46, A:Y47, A:S48, A:V49, A:L50, A:Y51, A:N52, A:S53, A:A54, A:S55, A:F56, A:S57, A:T58, A:F59, A:K60, A:C61, A:Y62, A:V64, A:S65, A:P66, A:T67, A:L69, A:N70, A:L72, A:C73, A:F74, A:T75, A:V77, A:Y78, A:A79, A:D80, A:S81, A:F82, A:V83, A:I84, A:R85, A:G86, A:D87, A:E88, A:V89, A:R90, A:Q91, A:I92, A:A93, A:P94, A:G95, A:Q96, A:T97, A:G98, A:K99, A:I100, A:A101, A:D102, A:Y103, A:N104, A:Y105, A:K106, A:L107, A:P108, A:D109, A:D110, A:F111, A:T112, A:G113, A:C114, A:V115, A:I116, A:A117, A:W118, A:N119, A:S120, A:N121, A:N122, A:L123, A:D124, A:S125, A:K126, A:V127, A:G128, A:G129, A:N130, A:Y131, A:N132, A:Y133, A:L134, A:Y135, A:R136, A:L137, A:F138, A:R139, A:K140, A:S141, A:N142, A:L143, A:K144, A:P145, A:F146, A:E147, A:R148, A:D149, A:I150, A:S151, A:T152, A:E153, A:I154, A:Y155, A:Q156, A:A157, A:G158, A:S159, A:T160, A:P161, A:C162, A:N163, A:G164, A:V165, A:E166, A:G167, A:F168, A:N169, A:C170, A:Y171, A:F172, A:P173, A:L174, A:Q175, A:S176, A:Y177, A:G178, A:F179, A:Q180, A:P181, A:T182, A:N183, A:G184, A:V185, A:G186, A:Y187, A:Q188, A:P189, A:Y190, A:R191, A:V192, A:V193, A:V194, A:L195, A:S196, A:F197, A:A204, A:T205, A:V206, A:C207, A:G208, A:P209, A:K210, A:K211, A:S212	193	0.769
**2**	C:P12, C:N13, C:I14, C:T15, C:N16, C:L17, C:C18, C:P19, C:F20, C:G21, C:E22, C:V23, C:F24, C:N25, C:A26, C:T27, C:R28, C:F29, C:A30, C:S31, C:V32, C:Y33, C:A34, C:W35, C:N36, C:R37, C:K38, C:R39, C:I40, C:S41, C:N42, C:C43, C:V44, C:A45, C:D46, C:Y47, C:S48, C:V49, C:L50, C:Y51, C:N52, C:S53, C:A54, C:S55, C:F56, C:S57, C:T58, C:F59, C:K60, C:N70, C:C73, C:F74, C:T75, C:N76, C:V77, C:Y78, C:A79, C:D80, C:S81, C:F82, C:V83, C:I84, C:R85, C:G86, C:D87, C:E88, C:V89, C:R90, C:Q91, C:I92, C:A93, C:P94, C:G95, C:Q96, C:T97, C:G98, C:K99, C:I100, C:A101, C:D102, C:Y103, C:N104, C:Y105, C:K106, C:L107, C:P108, C:D109, C:F111, C:C114, C:V115, C:I116, C:A117, C:W118, C:N119, C:S120, C:N121, C:N122, C:L123, C:D124, C:S125, C:K126, C:V127, C:G128, C:G129, C:N130, C:Y131, C:N132, C:Y133, C:L134, C:Y135, C:R136, C:L137, C:F138, C:R139, C:K140, C:S141, C:N142, C:L143, C:K144, C:P145, C:F146, C:E147, C:R148, C:D149, C:I150, C:S151, C:T152, C:E153, C:I154, C:Y155, C:Q156, C:A157, C:G158, C:S159, C:T160, C:P161, C:C162, C:N163, C:G164, C:V165, C:E166, C:G167, C:F168, C:N169, C:C170, C:Y171, C:F172, C:P173, C:L174, C:Q175, C:S176, C:Y177, C:G178, C:F179, C:Q180, C:P181, C:T182, C:N183, C:G184, C:V185, C:G186, C:Y187, C:Q188, C:P189, C:Y190, C:R191, C:V192, C:V193, C:V194, C:L195, C:S196, C:A204, C:T205, C:V206, C:C207, C:G208, C:P209, C:K210, C:K211, C:S212	180	0.761
**3**	B:I8, B:V9, B:R10, B:F11, B:P12, B:N13, B:I14, B:T15, B:N16, B:L17, B:C18, B:P19, B:F20, B:G21, B:E22, B:V23, B:F24, B:N25, B:A26, B:T27, B:R28, B:F29, B:A30, B:S31, B:V32, B:Y33, B:A34, B:W35, B:N36, B:R37, B:K38, B:R39, B:I40, B:S41, B:N42, B:C43, B:V44, B:A45, B:D46, B:Y47, B:S48, B:V49, B:L50, B:Y51, B:N52, B:S53, B:A54, B:S55, B:F56, B:S57, B:T58, B:N70, B:C73, B:F74, B:T75, B:V77, B:Y78, B:A79, B:D80, B:S81, B:F82, B:V83, B:I84, B:R85, B:G86, B:D87, B:E88, B:V89, B:I92, B:G98, B:K99, B:I100, B:N104, B:Y105, B:L107, B:I116, B:A117, B:W118, B:N119, B:S120, B:N121, B:N122, B:L123, B:D124, B:S125, B:K126, B:V127, B:G128, B:G129, B:N130, B:Y131, B:N132, B:Y133, B:L134, B:Y135, B:R136, B:L137, B:F138, B:R148, B:D149, B:I150, B:S151, B:T152, B:E153, B:I154, B:Y155, B:Q156, B:A157, B:G158, B:S159, B:T160, B:P161, B:C162, B:N163, B:G164, B:V165, B:E166, B:G167, B:F168, B:N169, B:C170, B:Y171, B:F172, B:P173, B:L174, B:Q175, B:S176, B:Y177, B:G178, B:F179, B:Q180, B:P181, B:T182, B:N183, B:G184, B:V185, B:G186, B:Y187, B:Q188, B:P189, B:Y190, B:R191, B:V192, B:V193, B:V194, B:L195, B:P203, B:A204, B:T205, B:V206, B:C207, B:G208, B:P209, B:K210, B:K211, B:S212, B:T213, B:N214, B:L215, B:V216, B:K217, B:N218	162	0.71
**4**	B:K309, B:T310, B:P311, B:P312, B:I313, B:K314, B:D315, B:F316	8	0.652
**5**	B:T252, B:Q253, B:T254, B:N255, B:R259, B:A260, B:R261, B:S262, B:V263, B:A264, B:S265, B:Q266	12	0.602
**6**	B:G317, B:G318, B:F319	3	0.56
**7**	C:G317, C:G318, C:F319	3	0.555
**8**	B:Y103, B:R139, B:K140, B:S141, B:N142	5	0.544
**9**	B:Q306, B:I307, B:Y308	3	0.537
**10**	B:P326, B:D327, B:P328, B:S329, B:K330, B:P331, B:S332, B:K333	8	0.53
**11**	A:T252, A:T254, A:N255, A:R259, A:A260, A:R261, A:S262, A:V263, A:A264	9	0.504

^a^ ElliPro predicts discontinuous antibody epitopes based on a protein antigen's 3D structure. ElliPro accepts an input protein structure in PDB format. ElliPro associates each predicted epitope with a score, defined as a protrusion index (PI) value averaged over epitope residues. In the method, the protein's 3D shape is approximated by a number of ellipsoids; thus, the ellipsoid with PI = 0.9 would include within 90% of the protein residues with 10% of the protein residues being outside of the ellipsoid, while the ellipsoid with PI = 0.8 would include 80% of residues with 20% being outside the ellipsoid.

**Table 5.** Results of T-Cell Epitope Prediction ^a^

Allele	#	Start	End	Length	Peptide	Core	Icore	Score
HLA-A*01:01	1	347	356	10	LADAGFIKQY	LADAGFIQY	LADAGFIKQY	0.840121
HLA-A*01:01	1	122	131	10	NLDSKVGGNY	NLDSVGGNY	NLDSKVGGNY	0.696259
HLA-A*01:01	1	227	236	10	IGAEHVNNSY	IAEHVNNSY	IGAEHVNNSY	0.485311
HLA-A*01:01	1	96	105	10	QTGKIADYNY	QTGKIADYY	QTGKIADYNY	0.437978
HLA-A*01:01	1	181	190	10	PTNGVGYQPY	PTNGVGYQY	PTNGVGYQPY	0.353345
HLA-B*56:01	1	17	26	10	LCPFGEVFNA	LPFGEVFNA	LCPFGEVFNA	0.451734
HLA-B*56:01	1	143	152	10	LKPFERDIST	LPFERDIST	LKPFERDIST	0.365267

^a^ The prediction method list box allows choosing from a number of MHC class I binding prediction methods: Artificial neural network (ANN), stabilized matrix method (SMM), SMM with a peptide: MHC binding energy covariance matrix (SMMPMBEC), scoring matrices derived from combinatorial peptide libraries (Comblib_Sidney2008), Consensus, NetMHCpan, NetMHCcons, PickPocket and NetMHCstabpan.

### 4.2. Confirmation of pET-28a Plasmid Containing RS Gene

**Figure 3.**
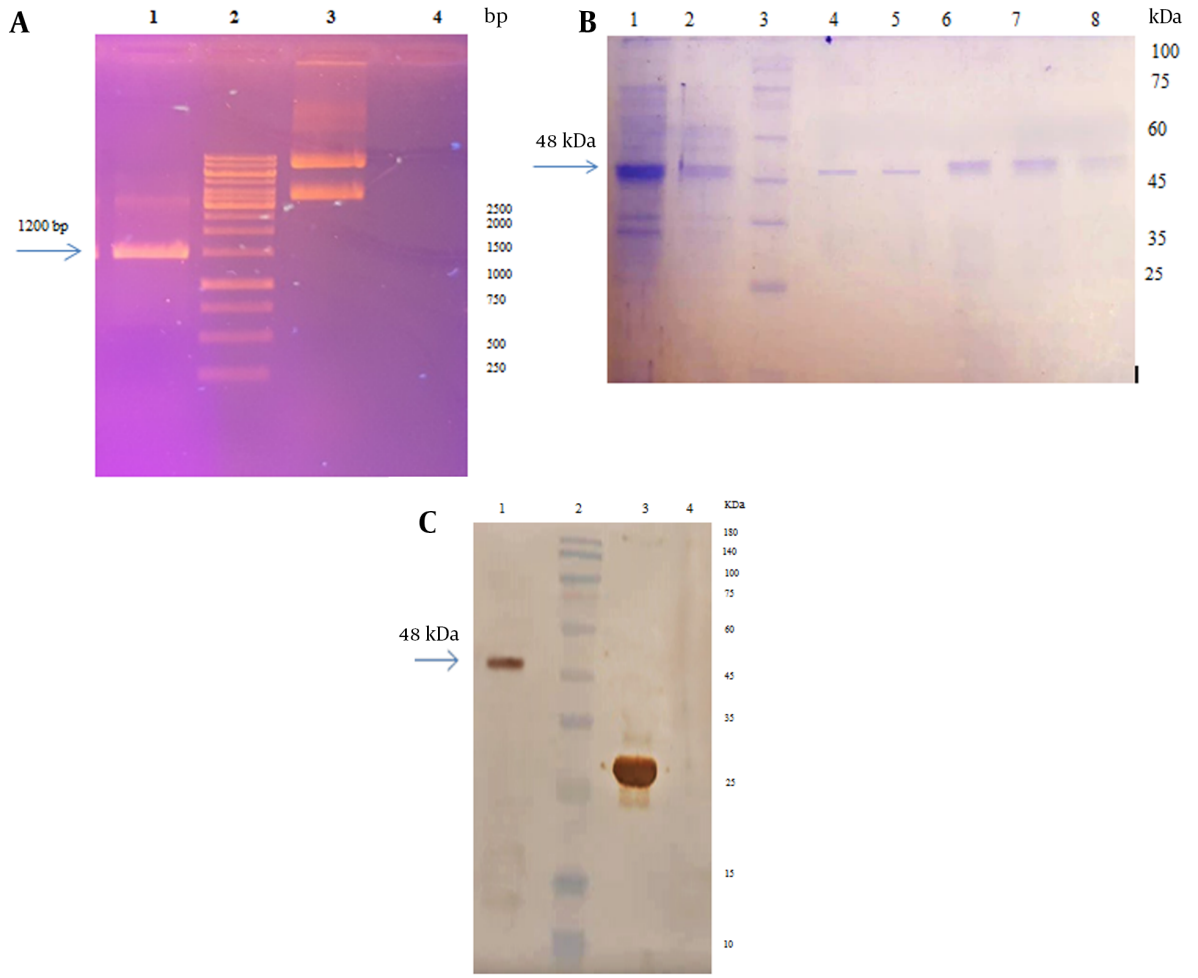
A, electrophoresis of pET28a- recombinant spike (RS) plasmid and PCR product on 1% agarose gel: Lane 1: PCR product with pET28a- RS by universal T7 primers, lane 2: DNA marker, lane 3: The extracted plasmid pET28a containing RS gene, lane 4: Negative control; B, electrophoresis of RS purified by the Ni-NTA column on 12% SDS-PAGE. Lane 1: Bacterial lysate, lane 2: Flow buffer, lane 3: Protein ladder, lane 4 - 8, purified protein (elution buffer); C, western blotting with the anti-His antibody. Lane 1: RS, lane 2: Protein ladder, lane 3: Positive control, lane 4: Negative control.

The results of plasmid extraction are depicted in Figure 3A. The PCR was performed using T7 universal primers to confirm the presence of the RS gene in the pET28a plasmid. A 1200 bp chimeric fragment cloned into pET-28a plasmid was observed on a 1% agarose gel (Appendix 1 in Supplementary File) (Figure 3A). 

### 4.3. Expression, Purification, and Confirmation of Chimeric Protein

The expression of the recombinant protein was evaluated using 12% SDS-PAGE, and the desired RS protein with a molecular weight of 48 kDa and a C-terminal 6x His-tag was detected. The recombinant protein was expressed as inclusion bodies (IB) and subsequently solubilized using 8 M urea (Figure 3B). The recombinant protein was purified through Ni-NTA affinity chromatography under denaturing conditions. The concentration of the purified spike protein, as determined by the Bradford method, was 213 µg/mL. The presence of the His-tag was confirmed through western blot analysis using anti-His-tag antibodies (Figure 3C). 

### 4.4. Determination of Serum IgG Titer

**Figure 4.**
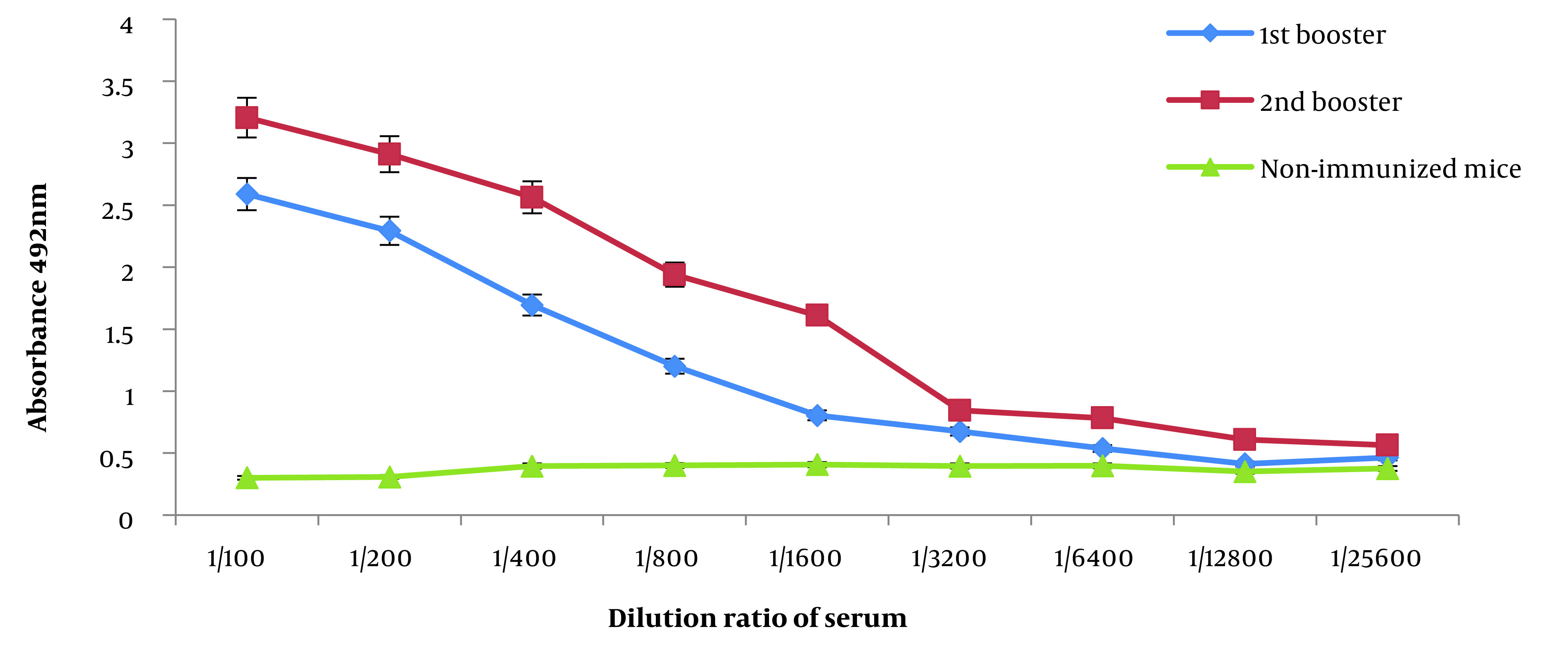
Immune response type analysis of mice immunized with recombinant spike (RS). Serum-specific IgG titers were taken from immunized mice. The detections were made by enzyme-linked immunosorbent assay (ELISA). Purified RS protein was applied as an antigen.

Blood samples were collected, and the levels of IgG antibodies were measured to determine antibody titers (Figure 4). The impact of the number of booster immunizations was assessed using repeated measurements. The results obtained from the indirect ELISA showed a significant increase in antibody titers with each subsequent booster dose administration (P < 0.05).

### 4.5. Detection of Inactivated SARS-CoV-2 Using Immunized Mice Sera

**Figure 5.**
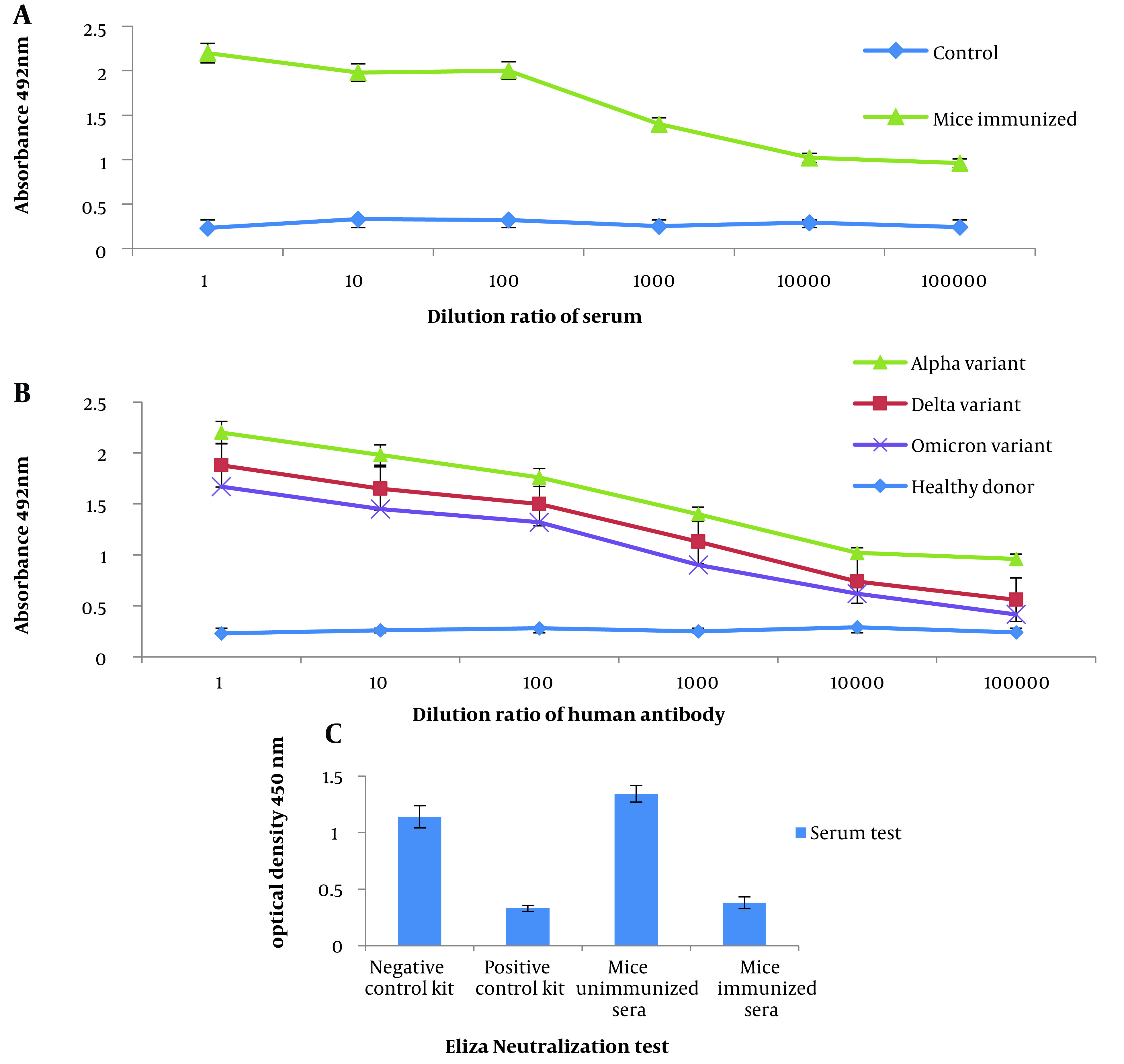
Evaluation of the potency of recombinant spike (RS). A, detection of the inactivated SARS-CoV-2 virus using the antibody of mice immunized with RS; B, diagnosis of RS by sera from patients infected with the alpha, delta, and omicron variants of SARS-CoV-2; C, ability to neutralize antibodies in immunized mice.

The results demonstrated that the serum from mice immunized with RS showed significant binding to the inactivated SARS-CoV-2 virus as an antigen compared to the control group. This finding was confirmed to be statistically significant (P < 0.05) through indirect ELISA (Figure 5A). 

### 4.6. Reactivity of RS in ELISA for SARS-CoV-2 Antibody Recognition

As shown in the diagram (Figure 5B), the strength of binding to antigen was higher in the alpha serum sample than in the delta, and the omicron sample was less susceptible to binding to RS as an antigen. Indirect ELISA confirmed the significance (P < 0.05) of the obtained result. With the dilution of the serum, the amount of binding of the antibody in the alpha, delta, and omicron variants to the RS protein as an antigen decreased, indicating the antibody's specificity to the antigen.

### 4.7. Characterization of the Sera from Vaccinated Mice by RS

The results suggest that the sera obtained from immunized mice exhibited a significant ability to inhibit the binding of the RBD to the ACE2 receptor on human cells compared to the sera from unimmunized mice (Figure 5C). 

## 5. Discussion

The omicron variant, characterized by numerous mutations in the Spike region, has emerged as a highly concerning variant with a significant impact on global health. It was first reported in South Africa and Botswana and has quickly spread due to its increased mutations and immune evasion capabilities, leading to a surge in new infections. This variant has been classified as a "variant of concern" due to its ability to evade neutralizing antibodies and reduce the effectiveness of existing vaccines. Understanding the activity of the spike protein region and the neutralizing antibody response is crucial for the development of effective vaccines. While some existing booster vaccines have shown effectiveness against omicron, others have proven ineffective. The continuous emergence of new variants with Spike region mutations necessitates the design of variant-specific vaccines to address future variants, which may require further analysis and research. Governments and health organizations worldwide should rely on past experiences and collaborate closely with the scientific community to deal with the current situation. This collaborative effort can lead to a better understanding of the virus and the development of therapies and strategies to overcome the COVID-19 pandemic.

Given the rise of the BA.2 sub-variant and the likelihood of further variations, proactive measures and ongoing research are essential to combat the evolving nature of the virus effectively. Recently, due to the time and expense associated with current approaches to vaccine development, computational vaccinology is often considered for the development of vaccines for a variety of diseases, particularly infectious diseases (31). Although the field of computational vaccinology is very young, many effective vaccines, such as the *Rickettsia prowazekii* vaccines (32), enterotoxigenic *Escherichia coli* (33), and many others, have been developed.

In the present study, we designed a subunit vaccine for cross-protection against variants type SARS-CoV-2, according to immunoinformatics and structural vaccinology strategies. All spike proteins were analyzed bioinformatically to make a chimeric protein. In a similar study, Wu et al. designed a subunit vaccine made of spike ectodomain protein SARS-CoV-2 (34). Fitzgerald et al. expressed surface glycoprotein fragments 319 - 640 in *E. coli* BL21 (DE3) (35). As shown in Figure 1A, the candidate vaccine consists of RBD, S1/S2 cleavage, and FP. The S1/S2 cleavage was used as a linker between RBD and FP. There are 3 to 5 epitope regions available in the RBD sequence for vaccine design (36). Due to the optimization performed on the RS gene and the RBD epitope, our sequence contained S1/S2 cleavage and FP region epitopes to stimulate the immune system. The final construct was analyzed for its structural, physicochemical, and immunogenicity properties using various bioinformatic tools. The results showed that the proposed vaccine is stable, insoluble, highly antigenic, and non-allergenic. From a practical point of view, to achieve an optimal level of protein expression in *E. coli*, some important parameters need to be optimized, such as the CAI, CFD, and GC content of the gene. The results confirmed that our proposed gene can be efficiently expressed in *E. coli* hosts. Choosing the right host for the expression and production of chimeric protein is one of the most important issues in research into subunit vaccines. The *E. coli* host is considered one of the most common and least expensive hosts for the production of recombinant proteins. However, this expression system cannot perform post-translational modifications of eukaryotic proteins (37). Many eukaryotic proteins maintain their three-dimensional structure and are fully biologically active in their non-glycosylated form. Therefore, they can be expressed in *E. coli* (38). The same is true for spike proteins, and despite the lack of post-translational changes (such as the addition of sugar molecules), it still retains their antigenic properties and can stimulate the immune system properly (39). The secondary and tertiary structures of RBD expressed in *E. coli* have largely been preserved without glycosylation (3). Properly forming disulfide bonds guarantees a protein's accurate and efficient structure (40). The spike protein has multiple disulfide bonds (41). Therefore, the *E. coli* Rosetta-gami (DE3) was selected, as it is a proper host for the spike expression due to uncommon codons (42) and the formation of target protein disulfide bonds in the bacterial cytoplasm (36).

The protein's three-dimensional structure plays a key role in the discontinuous (conformational) identification of B-cell epitopes and in protein-protein and protein-small molecule interactions (43). Therefore, the 3D structure of the designed vaccine was modeled using a Swiss model server. The structure quality and stability of the predicted model were assessed using the Ramachandran plot, ProSA Z-score, and quality factor. The results showed that a high-quality structure characterizes the predicted model. The input structure's Z-score was in the range of scores common for native proteins with similar sizes. The stability of RNA was tested; the lowest structure energy was -438.80 kcal. It is estimated that over 90% of B-cell epitopes are conformational. These epitopes play a key role in immunity by stimulating the production of antibodies (44). Therefore, the conformational epitope prediction was performed using the ElliPro server. As shown in Table 4, this suggests that the vaccine can induce strong humoral immunity against SARS-CoV-2. The prediction of discontinuous and linear epitopes from B-cells and T-cells revealed that these were scattered throughout the chimeric sequence. The results obtained indicate that the complexes tested have sufficient stability over time. On the other hand, while bioinformatics approaches have some advantages, such as time and cost efficiencies, due to some limitations, such as database limitations, low accuracy, considering interactions under ideal conditions, and lack of simulation of complex environments such as blood (45), in vitro and in vivo evaluations, it is necessary to confirm or reject the results.

In vitro, condition chimer protein was expressed in the *E. coli* Rosetta-gami (DE3) system. Proteins expressed by *E. coli* are insoluble. Insoluble proteins become natural and soluble when dialyzed with detergents (46). For more solubility protein, after adding IPTG, we reduced the temperature of the culture medium from 37°C to 16°C. The results of three doses of administration with pure RS protein showed that the target protein stimulated immune responses and produced high serum IgG antibody titers. This protein is a very good immunogenic with a high ability to stimulate the immune system. He et al. reported that the endpoint of the serum antibody titer with the highest dilution was 2.1 times higher than the optical absorbance of the control group (47). According to Figure 4, the endpoint of the serum antibody titer of mice immunized with our chimeric protein was 2.5-fold higher than the optical absorbance of the negative control. Therefore, it is likely that in the original model, in the first encounter with the antigen, a suitable amount of antibody is produced to fight the virus. This issue is especially important in immunization during pandemic conditions.

Finally, the ELISA method was used to investigate the presence of appropriate epitopes in the recombinant protein produced in the prokaryotic system and the effectiveness of this protein for cross-immunity with the COVID-19 virus. The antibody produced in the body of the test animal can detect the recombinant RS protein of about 48 kDa. In addition, the reactivity of the SARS-CoV-2 inactivated virus with sera from RS protein vaccinated mice suggested that the RS protein can present the same epitopes with the viral strain. Serological testing is a well-known technique for diagnosing SARS-CoV-2 infection (48). The findings showed the recognition of antibodies in the serum of people infected with alpha, delta, and omicron variants, via RS protein. As known, RS can be a suitable alternative to more complicated antigens in serological COVID-19 tests. The results showed that antibodies in vaccinated mice could interact between RBD and ACE2 receptors and affect neutralization against SARS-CoV-2. In our similar research, the expression of RS was checked in CHO (RS-CHO). It was more active regarding serological tests and epitope regions. Moreover, the production rate was reported to be 128 µg/mL (49). In RS-Ecoli, the production rate is equal to 213 µg/mL, and prokaryotic expression is of interest regarding low cost and access to facilities (50). It is suggested that in future studies, in addition to humoral immunity, cellular immunity be evaluated in mice immunized with the recombinant RS protein. One of the limitations of the current study is the absence of convenience and equipment to carry out the challenge test with the standard SARS-CoV-2 virus.

### 5.1. Conclusions

In brief, RS is assumed to be a potential subunit vaccine candidate for SARS-CoV-2, inducing humoral immune responses. The findings demonstrate the generation of S-protein recombinant as an antigen for serological ELISA testing, proposing a reasonable tool for detecting SARS-CoV-2 positive patients, which consequently could lead to a sensitive procedure for the timely detection of SARS-CoV-2 infection.

ijpr-22-1-137751-s001
