# Supplementary material for: Designing and Expression of Recombinant Chimeric Spike Protein from SARS-CoV-2 in Escherichia coli and Its Immunogenicity Assessment
Source: Iran J Pharm Res. 2023 Sep 10;22(1):e137751. doi: 10.5812/ijpr-137751 (PMC10728857; doi:10.5812/ijpr-137751)
Supplement: ijpr-22-1-137751-s001 [file ijpr-22-1-137751-s001.pdf]

Appendix 1

CTCGAGAAGCTTGCCACCATGGTGAGGGTGCAGCCCACCGAGTCCATCGTGAGGTTCCCCAACATCACCAACCT
GTGCCCCTTCGGCGAGGTGTTCAACGCCACCAGGTTCGCCTCCGTGTACGCCTGGAACAGGAAGAGGATCTCCAA
CTGCGTGGCCGACTACTCCGTGCTGTACAACTCCGCCTCCTTCTCCACCTTCAAGTGCTACGGCGTGTCCCCCACCA
AGCTGAACGACCTGTGCTTCACCAACGTGTACGCCGACTCCTTCGTGATCAGGGGCGACGAGGTGAGGCAGATC
GCCCCCGGCCAGACCGGCAAGATCGCCGACTACAACTACAAGCTGCCCGACGACTTCACCGGCTGCGTGATCGCC
TGGAACTCCAACAACCTGGACTCCAAGGTGGGCGGCAACTACAACTACCTGTACAGGCTGTTCAGGAAGTCCAAC
CTGAAGCCCTTCGAGAGGGACATCTCCACCGAGATCTACCAGGCCGGCTCCACCCCCTGCAACGGCGTGGAGGG
CTTCAACTGCTACTTCCCCCTGCAGTCCTACGGCTTCCAGCCCACCAACGGCGTGGGCTACCAGCCCTACAGGGTG
GTGGTGCTGTCCTTCGAGCTGCTGCACGCCCCCGCCACCGTGGTGTGCGGCCCCAAGAAGTCCACCAACCTGGTG
AAGAACAAGTGCGTGAACTTCGGCTGCCTGATCGGCGCCGAGCACGTGAACAACTCCTACGAGTGCGACATCCC
CATCGGCGCCGGCATCTGCGCCTCCTACCAGACCCAGACCAACTCCCCCAGGAGGGCCAGGTCCGTGGCCTCCCA
GTCCATCATCGCCTACACCATGTCCCTGGGCGCCGAGAACTCCGTGGCCTACAGGGCCCTGACCGGCATCGCCGT
GGAGCAGGACAAGAACACCCAGGAGGTGTTCGCCCAGGTGAAGCAGGTGAAGCAGATCTACAAGACCCCCCCC
ATCAAGGACTTCGGCGGCTTCAACTTCTCCCAGATCCTGCCCGACCCCTCCAAGCCCTCCAAGAGGTCCTTCATCG
AGGACCTGCTGTTCAACAAGGTGACCCTGGCCGACGCCGGCTTCATCAAGCAGTACGGCGACTGCCTGGGCGAC
ATCGCCGCCAGGGACCTGATCTGCGCCCAGAAGTTCAACGGCCTGCACCACCACCACCACCACTGA**GAATTC**
